# Understanding the mix of services for mental health care in urban DR Congo: a qualitative descriptive study

**DOI:** 10.1186/s12913-023-10219-x

**Published:** 2023-11-04

**Authors:** Erick Mukala Mayoyo, Bart Criel, Abdoulaye Sow, Yves Coppieters, Faustin Chenge

**Affiliations:** 1 School of Public Health, University of Lubumbashi, Lubumbashi, DR Congo; 2 School of Public Health, Université Libre de Bruxelles, Brussels, Belgium; 3 Department of Community Health, Institut Supérieur des Techniques Médicales de Kananga, Kananga, DR Congo; 4 National Mental Health Program, Ministry of Public Health, Hygiene and Prevention, Kinshasa, DR Congo; 5 Centre de Connaissances en Santé en RD Congo, Kinshasa, DR Congo; 6 Department of Public Health, Institute of Tropical Medicine, Antwerp, Belgium; 7 Faculty of Health Sciences and Techniques, Gamal Abdel Nasser University of Conakry, Conakry, Guinea

**Keywords:** Mix of services, Informal services, Traditional therapy services, Social services, Primary care services, Psychiatric services, Mental health workers, Qualitative descriptive study, Urban, Democratic Republic of the Congo

## Abstract

**Background:**

Mental health workers (MHWs) are exposed to conflicts of competence daily when performing tasks related to the provision of mental health services. This may be linked to a lack of understanding of their tasks as caregivers and providers. Furthermore, in most low-income settings, it is unclear how the available services are organized and coordinated to provide mental health care. To understand the above, this study aimed to identify the current mix of services for mental health care in the urban Democratic Republic of the Congo (DRC).

**Methods:**

A qualitative descriptive study was carried out in Lubumbashi from February to April 2021. We conducted 7 focus group discussions (FGDs) with 74 key informants (family members, primary care physicians, etc.) and 13 in-depth interviews (IDIs) with key informants (traditional healers, psychiatrists, etc.). We performed a qualitative content analysis, guided by an analytical framework, that led to the development of a comprehensive inventory of MHWs from the household level to specialized facilities, exploring their tasks in care delivery, identifying existing services, and defining their current organization.

**Results:**

Analysis of transcripts from the FGDs and IDIs showed that traditional healers and family caregivers are the leading providers in Lubumbashi. The exploration of the tasks performed by MHWs revealed that lifestyle, traditional therapies, psychotherapy, and medication are the main types of care offered/advised to patients. Active informal caregivers do not currently provide care corresponding to their competencies. The rare mental health specialists available do not presently recognize the tasks of primary care providers and informal caregivers in care delivery, and their contribution is considered marginal. We identified five types of services: informal services, traditional therapy services, social services, primary care services, and psychiatric services. Analyses pointed out an inversion of the ideal mix of these services.

**Conclusions:**

Our findings show a suboptimal mix of services for mental health and point to a clear lack of collaboration between MHWs. There is an urgent need to clearly define the tasks of MHWs, build the capacity of nonspecialists, shift mental health-related tasks to them, and raise awareness about collaborative care approaches.

**Supplementary Information:**

The online version contains supplementary material available at 10.1186/s12913-023-10219-x.

## Background

 Despite ongoing efforts to fill gaps in the management of mental disorders, very few countries in the world—particularly low- and middle-income countries (LMICs)—currently have an optimal mix of services for mental health care [1]. This mix of services can be defined as one that takes an integrated and comprehensive approach to the organization of services to address the informal and formal care needed by people with unmet mental health needs [2]. The management of mental disorders requires a mix of services such as self-care, informal care (e.g., shopping for patients, doing the laundry for them, “keeping an eye” on them), mental health care in primary care services, community-based mental health care (e.g., counseling, psychosocial education, monitoring of medication, sharing experiences and coping strategies, etc.), care in district psychiatric wards, and long-stay hospital psychiatric care [3]. However, in most LMICs, particularly African countries, the mix of mental health care services offered to patients is currently poorly understood and/or not implemented [1, 3, 4]. The World Health Organization (WHO) has provided countries with a pyramidal framework of an optimal mix of services to guide them in organizing services and ensuring access to informal and formal care for all [4]. The WHO also recommends that mental health services be established or transformed to promote self-care, create or strengthen informal community-based care delivery structures, integrate mental health care into primary health care (PHC) services, create community-based mental health services (CMHS), establish mental health services in district hospitals, and limit the number of psychiatric hospitals [3, 4]. Since then, this WHO framework (or model) has to the best of our knowledge never been used in the specific context of the Democratic Republic of the Congo (DRC) to identify who does what and at what level of the service pyramid to provide mental health care and psychosocial support to people with mental health problems. Furthermore, it appears from the literature review that the mix of services for mental health care is currently poorly known in the DRC, as it has never been studied.

For several decades, the responsibility of specialists (psychiatrists, psychologists, mental health nurse practitioners ‘MHNPs’, etc.) in the provision of mental health care has been widely recognized. However, in most LMICs, their number is very limited and grossly insufficient to cover all the mental health needs of populations facing many stressful and traumatic events. For example, in Africa, there are currently 0.9 mental health nurses, 0.1 psychiatrists, 0.1 psychologists, and 0.1 social workers per 100,000 people [5]. Thus, the gap in access to care remains of great concern due to a lack of financial resources but, above all, to a severe shortage of mental health professionals [6]. To fill this gap, the tasks of nonspecialist providers in providing such care have recently (again) been confirmed [7–9]. These include primary care providers (primary care physicians ‘PCPs’, nurse practitioners ‘NPs’, etc.), traditional healers, and other nonhealth actors such as patients themselves, educators, family members, community members, spiritual healers, also called church-based healers (i.e., priests, chaplains, lay people, pastors, religious counselors), etc., who are involved in the day-to-day management of mental health problems [10–12].

In the DRC, given that the coverage of mental health services is estimated at a dismal 5% [13], traditional medicine remains the main source of care for the population in both urban and rural areas [14]. Traditional African medicine can be defined in this study as the set of methods, practices, and rituals that involve the therapeutic use of plants, animal parts, minerals, and other medicines. African traditional medicine treatments are used for all sorts of mental health problems, but mainly for several mental illnesses manifested by delusions, withdrawals, and hallucinations, as well as for social problems [15–17].

In many African countries, including the DRC, a substantial part of people tends to believe that mental health problems have essentially supernatural causes such as the influence of ancestors, demonic possessions, transgression of taboos, witchcraft or human-made illness, bewitchment, etc., and that traditional therapy services are more effective than other types of services. This influences people’s knowledge and attitudes; therefore, traditional healers are often experts in this field. As a result, these traditional healers are often consulted either as a first resort for mental health problems, or even as the only ones able to provide appropriate mental health care. For instance, in a Nigerian survey, almost 45% of respondents stated that mental illnesses should first be treated in ‘the traditional way’ before going to hospital and that there should be no collaboration between formal health care providers and traditional healers for treating mental illnesses [18]. In addition to traditional medicine, people turn to other therapeutic practices such as fetishism, incantations, spiritual healing [19]. To treat mental health problems, traditional African healers combine medicinal plants, mystic-religious practices, and socio-cultural rituals. Although traditional and alternative treatments play an important role in meeting mental health needs, there is usually a tension, even controversy, between biomedical care providers and these informal service providers over the nature of indigenous traditional healing [19, 20].

In mental health care provision, nonhealth actors play a key role [21]. Family members are important caregivers who provide day-to-day care to relatives with mental health problems, take them to health facilities, counsel them, monitor their medications, take care of their financial needs, and much more [22, 23]. These different tasks described in the international literature remain unexplored in the Congolese context. Furthermore, the patient remains a central actor in the management of his or her mental illness and is responsible for self-care.

Managing mental health problems requires a more holistic approach that includes a range of informal, social, traditional, modern and other services, both hospital-based and community-based, whether in urban or rural, high-income or low-income settings. However, applying this approach can prove difficult when biomedical care providers strictly stick to hospital-based care, whereas informal caregivers advocate for community-based mental health services. Given the continuing controversy over the appropriate model of mental health services, a balanced approach including both community -based and hospital-based services is recommended, particularly in low-income settings where it is important to improve the coverage of primary mental health services with the help of specialists [24].

We assume that there is currently a range of services and mental health workers (MHWs) providing mental health care in urban DRC that can be involved in the process of integrating mental health into district health services. The WHO [25] defines MHWs as human resources working in the field of mental health, including professionals working in private and public mental health facilities, in private practice, as well as professionals and non-professionals working at community level. These may include psychiatrists, mental health nurses, social workers, psychologists, occupational therapists, other non-specialist nurses and doctors, officially recognized for these mental health tasks; but also, occasionally other healthcare staff, informal carers and even service users (who may also be peer carers), who work in the mental health sector, to provide both formal hospital and community-based and informal mental health services.

In the present research, we are focusing on urban DRC to identify the current mix of services for mental health care, especially the tasks that the actors perform and the organization and coordination of the services in which they provide care. When it comes to rural settings, as pointed out by Vergunst [26], formal mental health services are largely lacking in many sub-Saharan African countries. Mental health facilities tend indeed to be concentrated in towns and cities, depriving rural communities of specialists [27]. It would make sense to better describe and compare the perception of mental health problems and the mix of mental health services between urban and rural areas.

The results aim to provide policy-makers, health managers, and researchers with new insights on the current organizational mix of services for mental health in Lubumbashi; on the MHWs that are currently active; and on the tasks taken up by these MHWs, and in particular, nonspecialist providers and nonhealth actors involved in the provision of care. They will also inform the National Mental Health Program of the DRC and those of countries with similar profiles in the development of a strategy for integrating mental health into the primary care system, taking into account available services and actors.

## Methods

### Study setting

The study was conducted in urban DRC, more specifically in Lubumbashi, the second largest Congolese city, with an area of 747 km² and an estimated 2022 population of 2,695,000 [28]. The city of Lubumbashi, the capital of the province of Haut-Katanga in the southeast of the DRC, is part of the provincial health division of Haut-Katanga. This urban area is geographically divided into the following 9 health districts: Kamalondo, Kampemba, Katuba, Kenya, Kisanga, Lubumbashi, Mumbunda, Ruashi and Tshamilemba, and 107 unevenly distributed health areas [29].

The city has 69 hospitals, including those with more than 50 inpatient beds (the Provincial Hospital Janson Sendwe, the University Teaching Hospital of Lubumbashi, the *Cinquantenaire* Hospital, etc.) and those with fewer than 50 inpatient beds and an extensive network of 525 primary care facilities (including health centers) in both the private and public sectors [30]. The number of primary care facilities has increased dramatically over the years in a largely unplanned and uncontrolled way, especially in the private for-profit sector, which makes it difficult to provide accurate statistics.

According to a recent study, there are more than 110 identified traditional healers working in consultation offices in Lubumbashi [31], but the precise nature of their provision of mental health care remains poorly known. Some spiritual healers perform daily exorcism, incantation, and prayer practices to ‘heal’ or relieve the moral pain of patients with mental health problems who attend churches and prayer groups. In addition, family and community members also seem to play an important role in the management of mental health [32]. These tasks, therefore, will need to be further investigated in the context of this study.

### Study design

A qualitative descriptive study was conducted to collect data from February to April 2021. The chosen design allowed us to identify the mental health care services currently available and the tasks of the different stakeholders in the management of mental health problems. It also allowed us to address the lack of evidence on the mix of mental health care services in the study context.

### Study population and sampling

MHWs (considered key informants) participated in the study. They are all regularly involved in the provision of mental health (self)care in Lubumbashi. Depending on whether they were considered users or providers in the care process, participants were divided into two categories. The first category comprises mental health care users who are (ex-)patients, family members and community members. They have mental health needs that require care. However, although they are considered users, they are also occasional MHWs who practice self-care and/or provide basic mental health care and informal psychosocial support to their relatives in the community. The second category consisted of health care providers, namely ‘traditional’ health care providers (traditional healers and spiritual healers, so-called church-based healers), primary care providers (NPs and PCPs) and mental health professionals (psychiatrists, psychologists, MHNPs, social workers, etc.).

A purposive sample of key informants was gradually built up, with participants from 7 of the 9 health districts in Lubumbashi, until data saturation was achieved. In total, 98 potential participants were contacted individually. A message inviting them to participate in the study was delivered orally through community health workers who carry out health promotion activities during home visits.

Key informants were purposively selected according to their perceived experience with mental health and psychosocial support and their geographical area of residence and/or work. The aim was to include caregivers from different levels of the provincial health system, from the community to specialized services, as well as users of the various informal and formal care services in Lubumbashi. The participants met the following selection criteria: (i) be over 18 years old, (ii) declare a role in the provision or use of mental health care, (iii) speak French or Kiswahili, the two common and widely spoken languages in Lubumbashi, and (iv) voluntarily consent to participate in the study at no cost. For various reasons, 11 people (4 family members, 3 community members, 3 primary care physicians and 1 patient) declined to participate in the study. As they were part of the participant groups that were already sufficiently represented, one would not miss some important information from them. Ultimately, 87 key informants participated in the study (Table 1).

**Table 1:** Distribution of study participants in focus group discussions and individual interviews

Participant profiles	Focus group discussions (FGDs)		Interviews	Districts in which meetings were held
	Nb. of FGDs	Nb. of participants		
Family members	3	32	–	Tshamilemba, Kamalondo and Kenya
Community members	2	21	–	Kamalondo and Katuba
(Ex-)patients	–	–	4	Tshamilemba, Kenya and Lubumbashi
Traditional healers	–	–	2	Tshamilemba and Kisanga
Spiritual healers	–	–	2	Kamalondo and Kampemba
Nurse practitioners	1	11	–	Lubumbashi
Primary care physicians	1	10	–	Lubumbashi
Social workers	–	–	1	Katuba
Psychologists	–	–	2	Kisanga and Kenya
Mental health nurse practitioners	–	–	1	Lubumbashi
Psychiatrists	–	–	1	Lubumbashi
**Total**	7	74	13	

### Data collection methods and tools

We used focus group discussions (FGDs) and in-depth interviews (IDIs) to collect data. An interview guide, appended as supplementary file (Text S1), was used in both FGDs and IDIs. This guide with open-ended questions, developed based on the WHO model [4], was used as a data collection tool. The WHO model is a pyramid of services organization describing a so-called ‘optimal’ mix of health and social services for mental health. It promotes the idea that the most expensive and least necessary services should be reduced, while those that are most needed should be increased and provided at relatively low cost. Self-care is primarily provided at the local level and is present in all other services and at all levels of the pyramid [3, 4].

This tool addressed the following four predefined categories: (i) currently active MHWs; (ii) the tasks of MHWs in care delivery; (iii) existing services for mental health care; and (iv) the current mix (or organization) of services for mental health care and psychosocial support.

It was pretested with a sample of 7 key informants who participated in both an FGD and IDIs. These key informants were from Ruashi, a district that was not included in the study. This allowed us to ensure that the questions were clearly formulated and understandable, and no significant changes were made.

### Data collection

#### Conducting focus group discussions

We conducted 7 FGDs with key informants according to their profile, for instance, care users (family members and community members) and primary care providers (NPs and PCPs). FGD participants were homogeneously grouped among themselves (i.e., family members, PCPs), and the groups consisted of 10–12 participants. Groups were mixed internally according to gender and seniority to help avoid single-mindedness (or ‘groupthink’) that could lead to erroneous, one-sided data, and to uncover deeper insights [33] into the mental health tasks performed. The discussions took place from Monday to Saturday, between 8 a.m. and 6 p.m., at times when researchers and interviewees had good mobility. Depending on the preference of the discussants and to preserve privacy and confidentiality, the meetings took place either in a meeting room of the central office of the health district or at their own work office.

These FGDs were conducted face-to-face and lasted between 60 and 90 min, depending on the discussants. The moderator (principal investigator and first author) led the discussions. As these were internally heterogeneous FGDs, to guarantee free expression, the moderator took the time to channel tensions in constructive directions and ensured that the floor was distributed to both women and men, as well as juniors and seniors, to allow everyone to express their views. Two research assistants (young public health professionals, one female, and one male, trained in qualitative data collection techniques) audio-recorded the discussions with a dictaphone and took field notes. At the end of the discussions, the field notes and audio-recordings were transcribed verbatim and translated into French when necessary.

### Conducting in-depth interviews

IDIs were held for a minority of key informants in the city of Lubumbashi (e.g., psychiatrists and MHNPs) and for those who felt that participation in FGDs was not possible for any reason (e.g., traditional healers and patients). These face-to-face IDIs also lasted between 60 and 90 min depending on the eloquence of the interviewee. According to the preference of the participants and to preserve privacy and confidentiality, the IDIs took place either at the work offices or at the participants’ homes. The topic guide used for the FGDs was also used during the IDIs, with some questions oriented to the specific profile of the interviewee. During the IDIs, with the interviewee’s prior permission, the interviewer audio-recorded the exchanges using a dictaphone, and simultaneously took field notes. As with the FGDs, at the end of the IDIs, recordings and field notes were transcribed verbatim and translated into French whenever necessary.

### Data management

During data collection, the field notes and transcribed recordings were systematically reviewed before the subsequent FGDs and IDIs to allow for an adaptation of the data collection tool and the exploration of emerging concepts. Subsequently, the data collected were stored in a single digital file created and copied onto two computers and password protected. The principal investigator was responsible for data management and analysis. During transcription, data collected were anonymized and managed as such after the removal of the respondents’ identifiers. For all these data, the link between the code and the participants was permanently removed.

All transcripts were then captured and stored in an NVivo database for analysis. The quality and accuracy of the transcripts were checked by listening to all recordings (7 FGDs and 13 IDIs), and if necessary, the transcripts were corrected based on the respondent’s favorable opinion. This allowed credibility and validity to be examined by an independent evaluator.

### Data analysis

We conducted a qualitative content analysis; the unit of analysis was the services. The analysis of the data was guided by an analytical framework, appended as supplementary file (Figure S1), we developed, drawing inspiration from the WHO pyramid framework on the optimal mix of mental health services mentioned above. This analytical framework helped us to organize the data. Using an iterative categorization [34], the following four categories were selected: (i) actors (MHWs or stakeholders); (ii) tasks of these actors; (iii) existing services; and (iv) service organization.

The analysis was performed using NVivo 12. We proceeded to coding, the clustering of categories and subcategories predefined in the analytical framework and the extraction of verbatim passages. To minimize information bias, a triangulation of statements from specialists, nonspecialists and users was performed. To facilitate understanding of the current mix of services described in this study, we grouped the results into the four categories mentioned above.

### Ethical considerations

This study is part of a larger doctoral research project whose protocol was approved by both the Medical Ethics Committee of the University of Lubumbashi (UNILU/CEM/034/2021) and by the Institutional Review Board of the Institute of Tropical Medicine in Antwerp (IRB/RR/AC/187/1468a/21). Participation in this study was free and voluntary. Before participating in the study, all key informants signed the free and informed consent form themselves or by proxy (for those who could not read or write). They had the right to refuse and withdraw from the research without explanation. If they withdrew, we ensured that their identification data would be removed from the software. The researchers took care to keep all information provided in the discussions confidential. All those who participated in the discussions were also asked not to disclose the information that was shared.

All recordings were auditioned with the participant(s) and the groups involved to ensure the credibility of the data. These data were pseudonymized and then anonymized during computer processing before being exported to the analysis software. As the data were not very sensitive, it was agreed that it would be kept for a maximum of three years and then deleted.

## Results

Four main discrete but interrelated themes emerged from the data analysis: (i) MHWs currently active in the city of Lubumbashi, (ii) MHWs’ care tasks, (iii) Existing services for mental health care and psychosocial support, and (iv) The organization of these services.

### Currently active mental health workers

Participants in the FGDs and IDIs indicated that when they considered the mental disorders to be of supernatural or magic-religious origin, patients usually consult traditional healers (sometimes called diviners or marabouts) and/or spiritual healers. Family members participating in a FGD described this as follows:

*Mental illnesses are usually due to diabolical, evil spirits or bewitchments by witch doctors present in our families or society; because sometimes you are just sick without understanding why; depending on our beliefs or faith, we consult recognized traditional healers or our pastor or priest to cure and/or deliver us*. (FGD, family members, 22–49 years old)

In addition, the community members stated the following:

*Sometimes psychic disorders are due to disappointments by loved ones in the family or society. Sometimes they are of mystical origin. In some cases, the cause is natural. So depending on the case, the family can help (treat) the sick brother or sister themselves, as well as taking them to a traditional healer or a pastor or taking them straight to the hospital to consult doctors, NPs, and even psychologists*. (FGD, community members, 24–61 years old)

One patient reported the following:

*For my illness here (meaning the mental disorder), I have already consulted several people. I went to our priest for deliverance sessions. He, after doing so, recommended I consult a nurse at the health center Y, the latter after his consultation, advised me to see doctor K. I was followed by this doctor for 8 months, without success. The family took me to a traditional healer; there was some change. But when I relapsed, we went to a psychiatrist at the hospital, he is the one who is treating me well so far*. (IDI, patient, male, 48 years)

According to the mental health professionals we met, there are many active and nonactive MHWs in Lubumbashi. For those who are active, some provide care in the informal sector and others in formal health facilities. One mental health specialist said:

*Here there are several providers of psychiatric treatment and psychosocial care. I can mention: ourselves (psychiatrists), clinical psychologists, MHNPs, PCPs, social workers, nonspecialist doctors and NPs working in general hospitals and health centers. I can also add family members […]. Yes, people also consult traditional healers, even their church leaders… Other actors such as occupational therapists, socioanthropologists, community health workers, etc., are not currently active in mental health in Lubumbashi*. (IDI, psychiatrist, female, 2 years’ seniority)

In an IDI, another mental health professional, who also has a teaching position in a school of nursing, stated:

*There are other mental health providers, such as mental health technicians, patients, (ex-)patients (who act as peer helpers), community members, and supposedly ‘nonpatients’… There is a need to categorize them, depending on whether they are caring in the community, in general hospitals, or in specialized hospitals*. (IDI, MHNP, male, 12 years’ seniority)

**Fig. 1:**
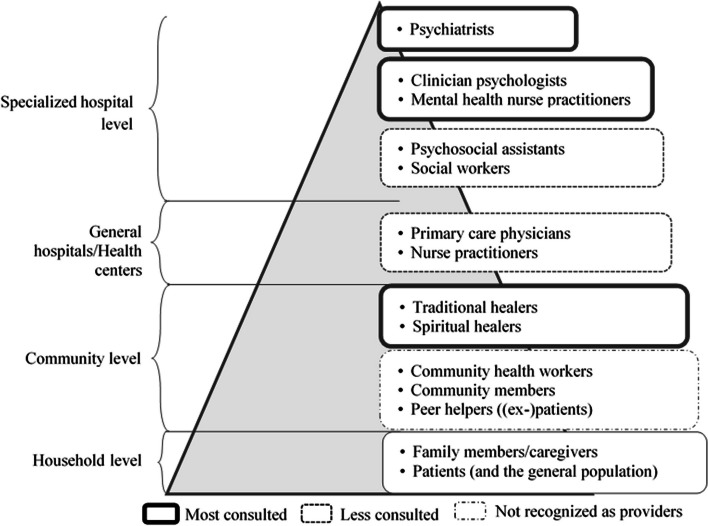
Pyramid of mental health workers in Lubumbashi (DRC) in 2021

All the stakeholders mentioned were positioned in the following pyramid of MHWs, indicating whether they provided care (Fig. 1).

### Tasks of mental health workers in care provision

Participants described the tasks they perform in managing mental health. Regarding self-care, one household member with a mental health problem said.

*My role is to accept my illness and keep hope for the future, to adhere to the treatment that has been prescribed to me; to follow the recommended lifestyle […]. I have to follow a healthy lifestyle and the treatment prescribed by the doctor […]. I seek the traditional remedy that has been recommended to me. Whenever I feel that something is wrong, I take it, and it gives me relief.* (IDI, patient, male, 48 years old)

One participant explained that when patients recover after being followed in the hospital, they continue to follow medical prescriptions but also act as peer helpers for others. He stated it as follows:

*When I come back to the community, I have the responsibility to respect good life hygiene, to follow the follow-up treatment, to respect the lifestyle advised by the caregivers and specialists, to help others who have the same problem as me, […] that is to say, to explain the mental health problems to others so that they can benefit from social support.* (IDI, ex-patient, male, 51 years old)

Most participants indicated that the tasks involved in caring for people with mental health problems (Table 2). Family members who participated in a FGD stated the following:

**Table 2:** Description of the tasks and services currently offered by MHWs when caring for a person with mental health problems

**Patient self-care** Respecting a healthy lifestyle; Self-medicate if symptoms are suspected; Seek treatment at home or in the hospital; Accept illness and hope for the future; Peer support when returning to the general population; Aftercare follow-up	
**Services provided by family members** Accompany patients to the hospital Purchase medication for patients and help them comply with medical prescriptions. Monitor patients’ clinical progress Screen brothers for mental health problems Provide transportation for the patient Look after the patient Paying medical bills Providing family support for patients	**Services provided by community members** Advise on the choice of care provider Raise awareness and resolve family conflicts Sensitize patients to going to the hospital Search for sociocultural causes of illness Organize meetings to make decisions about patients Make social support visits to patients Seeking out patients in the community
**Services provided by traditional healers** Detecting mental illness and its root causes Administer phytotherapy Perform incantations, rituals, incisions, excisions and scarifications Use of fetishes or black magic to heal the sick Bringing moral comfort Performing magic rituals Obvious treatment Use of palm oil Invocation to cemeteries	**Services provided by spiritual healers** Praying for patients Visiting the sick in the hospital Spiritual accompaniment for the sick Organize youth and prayer sessions and soul-cure sessions Teach the Word of God to the sick Cast out demons before sending the sick to the hospital Use of olive anointing oil Search for the spiritual cause of the illness
**Services provided by primary care providers** Assessing a person’s mental state Decide whether or not to refer the patient	**Services provided by mental health professionals (all disciplines)** Examine the patients carefully Medication (prescribing the right drugs) Psychotherapy Provide psychosocial support Support patients Conducting mental relaxation sessions Decide whether or not to cross-refer the patient

*As a family, we pray for our patients; we provide moral support; we accompany them to the hospital; we buy medicines for the patients; we monitor their clinical progress; we help identify mental health symptoms. We provide transport to the hospital.* (FGD, family members, 35–58 years old)

Still on informal family-based mental health care, another family member who participated in the FGDs stated that they performed different tasks as follows:

*We are usually the family caregivers when we take her to the hospital. Our tasks are to be close to the patient to support him, accompany him in taking medicines; pay the bills of care; have the right attitude towards the patient; maintain the hygiene of the patient; to prepare food for the patient and to psychological therapy.* (FGD, family members, 22–49 years old)

Community members contribute by advising and accompanying patients or their families in accessing care. As they stated below:

*Community leaders advise the family to accept the patient and explain how to take the products*. *If needed, we sensitize the community to avoid hitting, stigmatizing, or discriminating against patients*. (FGD, community members, 24–61 years old)

Box 1. Description of the tasks and services currently offered by MHWs when caring for a person with mental health problems.

For some FGD participants, while pastors pray for the mentally ill, traditional healers are instead described as practicing occult medicine for the benefit of individuals who have transgressed taboos, been bewitched and/or possessed by evil ancestral spirits. In addition to moral support, traditional-modern care is administered by healers who specialize in this care.

*The pastor prays for the sick and provides him moral support. But he has to ask the hospital for time to start praying for the sick.* (FGD, community members, 31–52 years old)

A spiritual healer who we met in an IDI indicated that the main task of faith healers is to accompany people spiritually and morally. He stated that.

*Our mission is to pray for the patients, to ensure the visit of the sick person to the hospital, to accompany the patient spiritually, to organize soul healing sessions and psychotherapies, to teach the word of God to the sick person, to cast out demons before sending the patient to the hospital.* (IDI, spiritual healer, pastor, 4 years’ seniority)

Other participants interviewed at the community level said:

*The traditional healer after doing his ceremonies (incantation, scarification…), gives remedies based on plants, animal skins, and many other things. He organizes sessions of traditional therapies and psychotherapies.* (FGD, community members, 24–61 years old)

When asked in IDIs, traditional healers stated that they perform tasks in the detection and treatment of mental disorders, especially traditional therapies and psychotherapies. One of them stated that.

*Our tasks consist of finding (e.g., diagnosis) the disease that affects the person, organizing sessions of psychotherapies and traditional therapies by giving him or her herbal and other remedies… Depending on the specialization of each of us, other colleagues perform incisions, excisions, and scarification… Fetishes, black magic, or other rituals can also be used to heal the sick.* (IDI, traditional healer, 16 years’ seniority)

Primary care providers stated that they are not, at present, skilled in carrying out the tasks of MHWs. PCPs from health centers and general hospitals said:

*Our tasks should be to identify a psychiatric disorder, prescribe medication such as tranquillizers to calm the patient with a mild case and then transfer to a specialist. But, currently we don’t do this, because of limited capacity.* (FGD, PCPs, 2–12 years’ seniority)

Nurse practitioners working in versatile primary care services do not currently appear to perform tasks required of them in the provision of mental health care. They stated that:

*Normally, our tasks are to give nursing care, administer prescribed medication, interact with the patient if he or she is not aggressive, and strengthen him or her with advice… But, at present, we are not doing so because of a lack of expertise.* (FGD, NPs, 3–15 years’ seniority)

A social worker who is employed in a local neuropsychiatric ward said:

*Our main contribution to care is to organize social surveys for diagnosis and social reintegration.* (IDI, social worker, male, 6 years’ seniority)

In contrast, a psychologist working in a nonprofit clinic described his tasks in providing advanced psychological interventions to individuals in distress:

*My tasks are to provide psychological support to people in psychological distress. I offer advanced psychological interventions, such as supportive psychotherapy, in case of psycho-trauma.* (IDI, psychologist, male, 3 years’ seniority)

One mental health professional described his tasks as follows:

*In this city, I perform neuropsychiatric consultations, prescribe psychotropic drugs, and advanced nursing care. I perform all the essential tasks of neuropsychiatry in the absence of the psychiatrist. I counsel patients on their lifestyle, do psychotherapy, etc.* (IDI, MHNP, male, 12 years’ seniority)

One interviewed psychiatrist described her clinical responsibilities as follows:

*My tasks are to assess the mental state of the patients thoroughly, to establish diagnosis, prescribe any medication (*i.e., *prescribe the appropriate drugs) and provide medical follow-up for complicated cases. I carry out psychotherapy, provide psychosocial support, conduct mental relaxation sessions, and decide whether or not to cross-refer the patient. In short, I am in charge of all cases that require complex psychiatric interventions.* (IDI, psychiatrist, female, 2 years’ seniority)

### Existing services for mental health care

The IDIs and FGDs conducted show that there are informal and formal health and social services in Lubumbashi that offer mental health care to the population. However, among these services, the statistics of those that provide mental health care are very limited.

The PCPs working in primary care services (health centers and general hospitals) interviewed mentioned some of the following health facilities:

*The recognized psychiatric hospitals are the Neuropsychiatric Center ‘Docteur Joseph Guillain’ in Lubumbashi and the Department of Psychiatry at the University Teaching Hospital of Lubumbashi. They offer highly specialized modern treatments as well as curative therapies based on specialized drugs. The treatment is multidisciplinary…* (FGD, PCPs, 2–12 years’ seniority).

One mental health professional working in a psychiatric facility mentioned only that center and the services it provides:

*At the Neuropsychiatric Center ‘Docteur Joseph Guillain’, medical and psychosocial care services are organized, including support meetings, psychosocial support interventions, family mediation, psychoeducation, psychosocial rehabilitation, and psychological and clinical follow-up. I also know of a private faith-based polyclinic that offers outpatient psychiatric care to patients. This is Afia Don Bosco.* (IDI, MHNP, male, 12 years’ seniority)

Additionally, NPs from primary care services cited one health facility and listed those services provided there, as follows:

*At the Provincial Hospital Janson Sendwe, there is a psychiatric ward that provides psychiatric care, but its capacity is very limited.* (FGD, NPs, 3–15 years’ seniority)

The transcripts analyzed revealed that the existing informal services delivering mental health care in Lubumbashi are neither listed nor mapped. Participants interviewed said the following:

*There are several churches in the city where the patients are prayed for and the word of God is preached to them. Priests and pastors provide spiritual support through prayer sessions, introspection, and moral support. Prayer over the water the sick person drinks; identification of sins that can affect mental health (fornication, theft, …). But we cannot know how many churches specifically do this.* (FGD, community members, 31–52 years old)

One mental health professional in an IDI, however, stated the following:

*Yes, traditional healers have their traditional therapy services where they treat the sick person. They give herbal remedies, and powders to apply […]. They treat either by incantations, scarification, deliverance, the use of olive oil, an invocation to the cemeteries, or magical rituals depending on their diagnosis or the cases to be treated… There are also social services that provide mental health care… Without a specific survey, it is not possible to know the exact statistics of all these services…* (IDI, MHNP, male, 12 years’ seniority).

### Organization of services for mental health care and psychosocial support

**Fig. 2:**
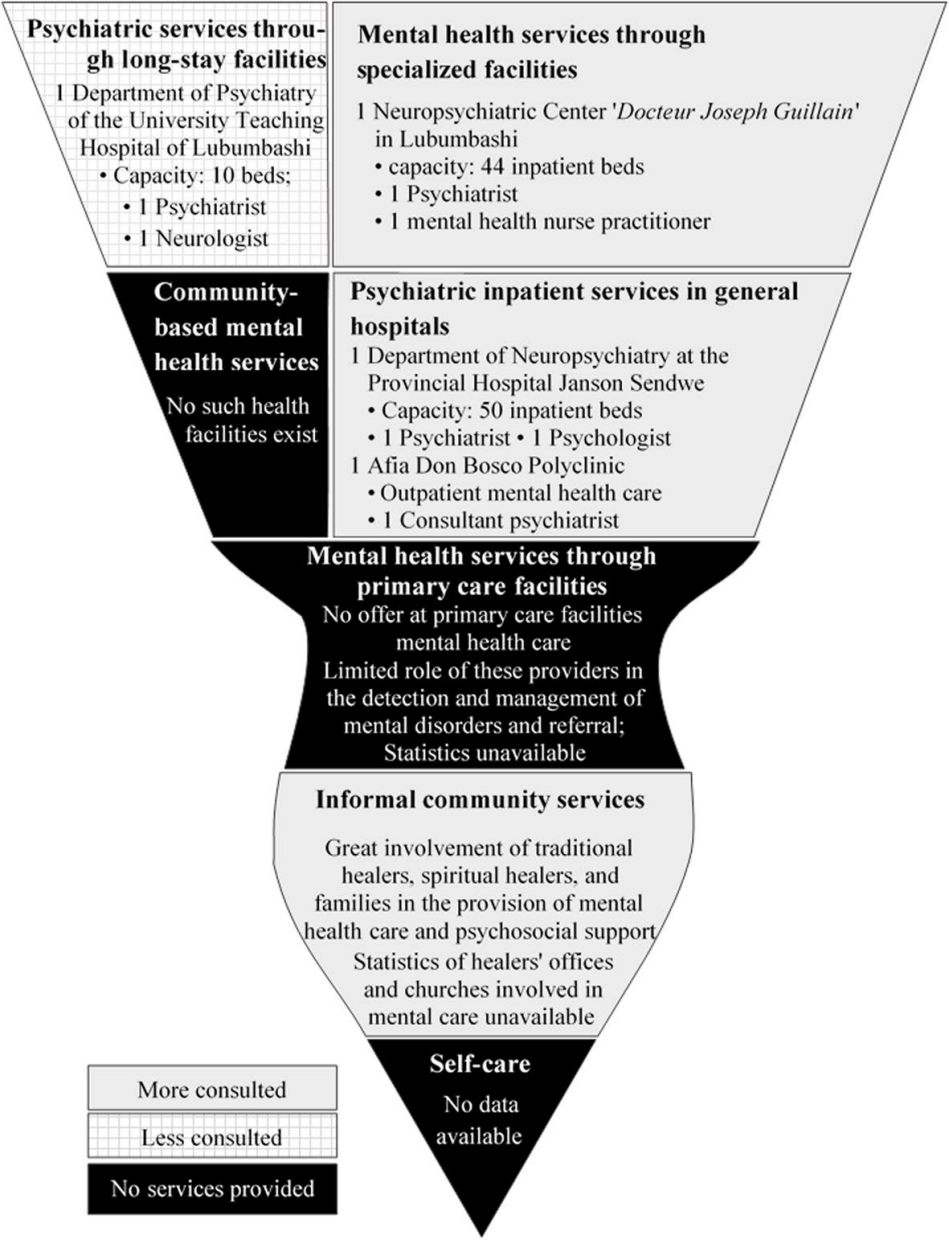
Mix of mental health services in the city of Lubumbashi (DRC), 2021

The transcript analysis showed that the mix of health and social services available for mental health care and psychosocial support in Lubumbashi is currently suboptimal (Fig. 2).

A provider participating in the IDI indicated that the current mix of services creates a disruption in terms of patient referral and counter-referral. He stated it as follows:

*There is a disorganization of services because someone can leave home and go directly to a psychiatric hospital without going through a health center or a general hospital, and after recovery, they are returned directly to their home. It becomes difficult to follow up on cases in aftercare. It is also with reason because all these health centers do not provide psychiatric care*. (IDI, MHNP, male, 12 years’ seniority)

Some participants stated that collaboration between MHWs currently hardly exists because these workers actually do not know each other. They are unaware of who can do what, where and when, wishing that something would be done to optimize collaboration. They stated in an FGD:

*The collaboration between the actors in the care of the mentally ill is not good because they do not know each other, and there is also a reluctance between the actors*. (FGD, family members, 35–58 years old)

Some participants in the study stated that collaboration between MHWs is not currently good, in particular because these workers do not know each other, i.e., they do not know who can do what, where and when. They want something to be done to optimize this collaboration. Along with the family members, the community members stated the following:

*There is a serious problem of collaboration between these different caregivers because when a mental health problem is identified in the city, the first thing is to go to the church to pray because they say that they are demons […]; when they realize that it is not possible to heal, they come to the hospital. The services are not well organized; there is no hierarchy*. (FGD, community members, 24–61 years old)

NPs interviewed during a FGD at the general hospital stated that the arrangements for collaboration were not well defined, which explained the late attendance of patients at care settings. They put it this way:

*The patient only comes to the hospital when the case is serious, after unsuccessful self-medication or after having seen a traditional healer or a pastor. But the modalities of a clear collaboration are not yet predefined because we do not know their limits*. (FGD, NPs, 3–15 years’ seniority)

Other participants recommended the involvement of various MHWs in the management of mental health problems, and insisted on the need for sound collaboration between these different stakeholders involved in service provision. They stated it as follows:

*I would prefer that all the stakeholders involved in the treatment of mental illnesses be listed so that they can begin to collaborate, given that the management of these pathologies requires the involvement of several actors. It must be recognized that the competencies of each person may be limited to what they know and depend on the causes of the illness*. (FGD, community members, 24–61 years old)

Several actors who participated in a FGD emphasized multidisciplinary interventions. Recognizing the multifactorial origins of mental health problems, they encouraged the combining of several types of services, such as psychiatric, traditional, social, informal and others. They stated that:

*Neuropsychiatric diseases are at the crossroads of several scientific and nonscientific disciplines. For the well-being of the patient, these different disciplines must collaborate. Healing must be total: physical or organic, mental, and spiritual*. (FGD, community members, 31–52 years old)

Some respondents believed that a collaboration between modern health care professionals and spiritual healers would be possible. Others, on the contrary, have doubts and state that such a collaboration is difficult. One of the spiritual healers we met in the IDIs said:

*Collaboration between us (e.g., religious leaders, pastors) and modern providers, yes, it must exist but not with traditional healers. We will never collaborate with the latter because what they do is in a way that is in contradiction with the divine word*. (IDI, spiritual healer, pastor, 10 years’ seniority)

A MHNP we met spoke along the same lines as the spiritual healer. We have heard that the collaboration exists between health professionals practicing modern medicine, and sometimes with spiritual healers. He stated the following:

*Collaboration is very important and exists, especially between the health care team (psycho-clinician, psychiatrist, and others) and the spiritual healers. But with the traditional healer, I am reserved because I have never seen him come to our Center, except for the spiritual healers*. (IDI, MHNP, 11 years’ seniority)

We noted that each category of services operates in isolation. PCPs in a FGD stated that traditional healers and others should bring all patients to specialized facilities, as they are the only ones authorized to provide quality psychiatric care. PCPs participating in a FGD stated the following:

*Ask the traditional healers and pastors to release the patients they keep in their practices and churches. Those who play the biggest role are medically educated health care workers*. (FGD, PCPs, 2–11 years’ seniority)

On the other hand, spiritual healers feel that they have a substantial role to play compared to other categories of actors, given the supernatural causes of mental illness.

*We, the pastors, have a big role in the management of mental illness compared to other actors. If the patient is not freed from his charges (*i.e., *evil spirits), medical treatment will fail*. (FGD, spiritual healer, 4 years’ seniority)

Traditional healers consider themselves more competent in the field of mental health care. They insisted that most mental illnesses have non-biological origins, and should therefore be treated by traditional medicine.

*Everyone knows that mental illnesses have a supernatural origin. Pharmaceutical drugs help to calm the crises, but it is ancestral medicine that heals the disorders in the long term*. (FGD, traditional healer, 4 years’ seniority)

The care users interestingly formulate a different view. According to them, every provider—whatever its nature—can contribute to the management of mental illnesses. They stated it in the following terms:

*I am a witness myself, I had a mental health problem and I got my health back at prayer*. (IDI, ex-patient, male, 51 years old)

However, family members acknowledge the merits of traditional medicine. One of them told a FGD that he himself had seen his own brother cured by a traditional healer. He stated the following:

*My brother had a mental health problem; it was a traditional healer who cured him*. (FGD, family members, 35–58 years old)

Users stated in one of the FGDs that each single actor, within the limits of his or her competence, can contribute to mental health care. They stated that if each caregiver recognized his or her limits, this would foster collaboration. They put it like this:

*Honesty is also about recognizing your limitations. If you cannot do something, you have to take it to the one who is capable according to the gift of everyone*. (FGD, family members, 22–49 years old)

## Discussion

This study, which aimed to identify the current mix of services for mental health care in urban DRC to understand the tasks performed by MHWs and the organization of services, reveals a complex situation of the mix of services for mental health in Lubumbashi. Its findings deserve special attention in the (re)organization of services in the implementation of the planned integration of mental health into the primary care system across the DRC and in other low-income settings with similar contexts.

### The organization of the existing services

By analyzing the organization of existing health and social services and the collaboration between MHWs, we found that there is no clear collaboration in the management of mental disorders between care providers trained and operating in a biomedical logic on the one hand and informal caregivers on the other, and the mix of these services is currently suboptimal and actually takes the shape of an inverted pyramid.

As highlighted by Nyame et al. [35], the main reason for the lack of effective collaboration is the persistence of mutual suspicion between the wide range of MHWs involved in the care of people with mental health problems. These researchers indicated that primary care providers fear that informal providers will harm service users, for example, through delays in care pathways and human rights violations (keeping patients in chains and exposure to weather vagaries, etc.). However, both service users and carers emphasized the inability of Western medical care providers to meet their mental health care needs, not least because of frequent shortages of psychotropic medication in health facilities. Effective collaboration will help provide more comprehensive and patient-centered care. There is a need to sensitize and educate all MHWs on the benefits of collaborative approaches.

The non-optimal mix of mental health care services in urban DRC described in this study, especially using the WHO’s pyramid framework, has to the best of our knowledge not been extensively explored in other African countries. In some African countries, such as South Sudan, there are no long-stay facilities or specialized mental health facilities, so the top level of the pyramid is absent [36].

When describing the organization of services for mental health and psychosocial support, the study revealed that the current mix of services is characterized by a lack of integration in terms of the flow of patients in the health system with a poorly organized referral and counterreferral system of patients. The WHO recommends reducing the number of psychiatric hospitals [3, 4]. Our study showed that the number of psychiatric hospitals is limited in Lubumbashi but also that their capacity remains insufficient to cover the needs of the whole population. In this context, a reduction in the number of existing psychiatric beds is not an option. On the contrary, it is necessary to increase the capacity of existing mental health facilities by a factor of 10 to reach the international standard [37]. The high use of the few existing mental health care facilities, the virtual absence of self-care and informal care services, and the limited number of mental health services in district hospitals would explain the observed pyramid inversion. The question is now how to match supply and demand.

### Existing services for mental health care and psychosocial support

At individual and household levels, we found that people lack knowledge of Western psychiatry and ‘modern’ mental health care practices. However, when faced with mental health problems, they may very well rely upon self-help and coping skills, and be resilient. At the community level, where stigmatization prevails [14], people with unmet mental health needs resort essentially to spiritual and traditional healers, as there are no community-based organizations, no school-based services, no youth clubs, no user associations, etc., taking care of mental health patients. At the primary care level, we found that no health centers or district hospitals are currently offering mental health care. Nor are there any statistics or mapping of services offering mental health care in Lubumbashi, such as informal services and CMHS. These concerns about statistics can generally only be addressed through quantitative surveys. At the secondary level, there is only one psychiatric inpatient service (the Department of Psychiatry) at the Provincial Hospital Janson Sendwe, with a capacity of 25 psychiatric inpatient beds, and one psychiatric outpatient mental health service at the Afia (Don Bosco) Polyclinic, a private, not-for-profit facility. At the tertiary level, there are two health facilities specialized in long-stay psychiatric care, namely, the Neuropsychiatric Center ‘*Docteur Joseph Guillain*’ with a capacity of 44 psychiatric inpatient beds and the Department of Psychiatry of the University Teaching Hospital of Lubumbashi with a capacity of 10 psychiatric inpatient beds. The latter remains, according to FGD and IDI participants, underutilized due to the high cost of care. This statement requires a quantitative study of mental health service utilization. Thus, in Lubumbashi, there is a severe shortage of psychiatric inpatient beds, with only 2.82 psychiatric inpatient beds per 100,000 inhabitants. This figure is far below the international standard, which ranges between 30 and 60 psychiatric beds per 100,000 inhabitants [37].

### Mental health workers’ care tasks

Regarding the actors involved in mental health care, participants cited a variety of MHWs, such as psychiatrists, clinical psychologists, MHNPs, PCPs, NPs, psychosocial workers, spiritual healers, family caregivers, and traditional healers. Knowing that there are many stakeholders could be a reason for satisfaction. However, in the last 5 years, in DRC, no data have been reported per 100,000 populations for key MHWs such as psychiatrists, nurses, social workers, psychologists, occupational therapists and other nonspecialized doctors working in the mental health sector [25]. Respondents, however, pointed out that some professional categories were not active because of a lack of required skills. The interviewed PCPs and NPs stated that they were not fulfilling their role adequately in mental health tasks such as screening, diagnosis, treatment, follow-up, referral of severe cases, and management of counterreferrals. This may be related to their low capacity in this area, as the initial training they received appears to be superficial and overly theoretical and does not allow them to properly diagnose, treat and refer only those cases that require services from specialists. However, the management of people with mental health conditions in general versatile healthcare settings helps reduce mental health-related stigma [8]. To achieve this, a capacity-building program for these primary care providers is needed to enable them to detect, diagnose, treat, and monitor these patients.

The results showed that community health workers are not recognized as mental health care providers. However, a study in Tshopo Province in the DRC indicated that community health workers provide health care, including mental health care, suggesting that task-shifting would work in their favor [38]. The first experience with mental health integration in the North Kivu province in the DRC showed that community health workers contributed significantly to mental health promotion through awareness-raising, community mobilization, follow-up of patients during home visits, and more [13].

### Mental health workers currently active

We found a significant lack of mental health professionals such as psychiatrists, clinical psychologists, and mental health nurses. Although the role of specialists has been widely recognized in the provision of care [8], these mental health professionals are sorely lacking in our context. As the WHO [5] notes, there are too few mental health professionals in Africa to cover the huge unmet mental health needs of all populations, as there is currently less than one specialist (all profiles) per 100,000 people.

Non-specialized health professionals, lay workers, affected persons, and family and community caregivers are capable, with brief training and appropriate supervision by mental health specialists, of detecting, diagnosing, treating, and monitoring people with mental disorders and reducing caregiver burden [39]. The lack of appropriate human resources has a negative impact on the provision of services, particularly those based on Western mental health practices. In this context, patients attend health facilities without any benefit for them. This unmet need for care can be a source of frustration, anger, and abandonment of the ‘modern’ care system when needed.

While our study shows that there are many MHWs, it highlights that traditional healers and family caregivers are currently the main care providers. Traditional healers can offer traditional therapies that are deemed to be effective, provided they are administered appropriately. In Africa, and particularly in the Eastern region, four main traditional therapies are generally administered by traditional healers: herbal medicine, surgery (incisions, scarification…), psychotherapy, and spiritual therapy [40]. In our context, the tasks of spiritual healing are carried out by spiritual healers (priests, lay pastors, imams, etc.). However, Congolese legislation recognizes the traditional healer as a health care provider *à part entière*, including mental health care. It defines him or her as ‘any individual who usually advises on methods of maintaining or improving health and treats human illness, physical or mental illness by faith and spiritual guidance or by means traditionally used by the community and believed to heal by aiding or stimulating nature’ and authorizes him or her to offer medical (herbal) care, psychotherapy, and spiritual care [41]. However, the majority of mental health professionals trained in a biomedical perspective still show strong skepticism or even outright disapproval of traditional healers [42] and opposition to a culture that trusts in spiritual healing [43]. In countries such as the DRC where religious demographics indicate that over 95% of the nearly 100 million Congolese attend places of worship [44] and thus retain faith in spiritual healing, rejecting the category of spiritual healers would contribute to social tensions between biomedical and psychosocial paradigms and possibly even the rejection of ‘modern’ mental health care by the faithful or believers. Furthermore, regarding traditional medicine treatments, clinical studies conducted in other African countries (Nigeria, Uganda, South Africa, etc.) have established that patients with mental disorders such as psychosis subjected to these treatments, alone or combined with biomedical treatments, recovered within a reasonable time [16, 45, 46]. Given that people trust these providers (i.e., traditional healers), one of the clever and deliberate strategies for expanding access to and use of mental health services could be to integrate these traditional healers into the science-based (mental) health care system, thereby increasing faith and trust in the system.

Family members act as caregivers. They accompany the patient to the hospital and encourage him or her to follow the care and take the prescribed medications, and they bring the patient to the hospital, where they usually act as a warden. The family also provides moral, spiritual, and financial support. In LMICs, where social protection is limited, financial support from the family is of great relevance. Because stigmatizing attitudes toward patients with mental disorders are highly visible in urban areas of the DRC [14], family caregivers of patients with mental illness may also be exposed to stigma. Among the strategies to reduce stigma for family caregivers, for instance, improving mental health literacy, education and coping strategies, promoting well-being, etc. [47], there is a need to seek and implement those that would be contextually most appropriate.

Our results showed that the mental health care system in the city of Lubumbashi is highly fragmented; each category of service operates in isolation; health professionals trained in the biomedical model feel they are better able to play a critical role in providing psychiatric care than traditional and spiritual healers. In addition, among biomedical providers, specialists feel they are in the best position to provide care compared to nonspecialists. Specialists recommend that nonspecialists and traditional healers refer their patients to specialized facilities as soon as possible, as they are not afraid of being overworked. Thus, the majority of MHWs are not willing to collaborate in providing care to patients. However, the complexity of the needs of people with mental health disorders requires a range of health care services offered by actors from a variety of disciplines and sectors in integrated services and coordinated systems of care to support their recovery, promote their well-being and optimize their social (re)integration. As van Rensburg and Brooke-Sumner [48] point out, given the complex care needs of people with mental health problems, no single professional category can claim to promote the full recovery of these patients. To enable recovery, a comprehensive (or biopsychosocial) multidisciplinary, inter- and multisectoral approach is more than recommended. However, we believe that traditional therapists (herbalists, spiritual healers, general practitioners, etc.), informal caregivers, primary care providers, and mental health professionals should only work together in the care of patients with mental disorders if they develop an understanding of the tasks of each professional category, trust each other, and agree to collaborate beyond their strict areas of biomedical, psychosocial and sociocultural expertise. This is an issue that deserves careful consideration.

### Strengths and limitations

This study, whose findings are based on primary data collected from a wide range of key stakeholders, has the benefit of informing decision-making on the reorganization and mix of mental health services and psychosocial support in the DRC. It may inspire other similarly profiled resource-limited settings. The study included a wide range of MHWs from both the formal and informal sectors as participants. The results, therefore, reflect different views and broader perspectives than other studies that have interviewed only (future) specialists, who are likely to be more knowledgeable about the issue [49].

However, this study did not include in its sample government decision-makers and health facility staff to determine how tasks are shifted to providers working in their health and social facilities and what they think of the current organization of services. Furthermore, this study does not provide details on the number of MHWs actually active in mental health across all categories or the number of social services involved in mental health management. A census can be initiated to quantify the active MHWs, as well as the community-based social organizations involved in mental health. The interviewers were physicians trained and practicing ‘modern’ Western medicine daily. They were generally perceived as defenders of the Western biomedical model. Given their specific social position, they were likely to exert some influence on the data collection process. However, the moderator, a mental health professional by background and, trained in multidisciplinary approaches to mental health care, helped both interviewers and interviewees to ensure that all views on mental health care, whether formal or informal, were important and legitimate. This contributed to minimize bias.

The choice of mixing focus groups according to characteristics such as gender and seniority is often debated by researchers because it is likely to influence the results. Furthermore, Femdal and Solbjør [50] stated that ‘*Mixed focus group interaction can make a valuable contribution to developing knowledge in the field of mental health service research*’. Given that the field of mental health care still is grossly underdeveloped in the DRC and that people still largely resort to informal mental health care services, it was deemed important to organize mixed FGDs, including men and women, juniors and seniors. The moderator ensured that (i) participants in the FGDs were people who knew each other, (ii) freedom of expression was guaranteed, and (iii) there were no people monopolizing the floor to deny the opportunity to express themselves.

However, although the moderator ensured that the floor was shared, tensions channeled and free expression encouraged, it is generally accepted that in non-Western countries such as the DRC, younger people may believe it is disrespectful to offer comments that differ from those stated by their elders or it may be considered unwise for women to argue with men [51]. Thus, by mixing senior and junior healthcare providers during heterogeneous FGDs, the latter would have felt uncomfortable providing information that contradicted what the seniors (whom they refer to as ‘masters’) had said. This might be one of the study’s limitations to be taken into account in future research.

## Conclusions

Our results show an inversion of the ideal mix of services for mental health in Lubumbashi. Optimizing the current mix of services, which is currently distributed in an inverted pyramid, will require integrating evidence-based mental health care into primary care settings, including self-care promotion, in a participatory design. Among the MHWs available in Lubumbashi, two main care providers (traditional healers and family caregivers) are currently very active in mental health. As there is no clear collaboration between MHWs in the management of mental disorders, there is a need to listen to the views of all stakeholders on the perceived benefits and constraints of collaborating in the provision of care [35], engage in a genuine dialogue, and sensitize stakeholders to offer psychiatric patients comprehensive and integrated care.

Five types of mental health services are available in the city of Lubumbashi. However, users mainly prefer to use traditional therapy services. Given the severe shortage of specialists in the DRC in general, and especially in Lubumbashi [52], improving mental health literacy, capacity building and task-shifting to primary care providers is essential. Moreover, providers such as traditional healers as well as family caregivers deserve wider recognition given their potential to reduce the workload of specialists.

As it was pointed out that the mental health landscape, and more in particular the organization of mental health care services, may differ between urban and rural areas [26], and given the fact that the DRC is currently engaged in a national dynamic of progressive integration of mental health into the primary care system, there is need to conduct similar study in rural DRC. In the long term, studies quantifying the available structures offering mental health services in Lubumbashi, and those measuring the utilization of mental health services may be needed.

### Supplementary Information

**Additional file 1: Text S1.** Interview Guide

**Additional file 2: Figure S1.** Analytical framework of the mix of mental health services

## Data Availability

All data generated or analyzed during this study are included in this published article and its supplementary information files.

## Abbreviations

CMHS: Community-based mental health services
DRC: Democratic Republic of the Congo
FGDs: Focus group discussions
IDIs: In-depth interviews
LMICs: Low- and middle-income countries
MHNPs: Mental health nurse practitioners
MHWs: Mental health workers
NPs: Nurse practitioners
PCPs: Primary care practitioners
PHC: Primary health care
WHO: World Health Organization
