# Supplementary material for: Understanding the mix of services for mental health care in urban DR Congo: a qualitative descriptive study
Source: BMC Health Serv Res. 2023 Nov 4;23:1206. doi: 10.1186/s12913-023-10219-x (PMC10625694; doi:10.1186/s12913-023-10219-x)
Supplement: Supplementary file 1 — Additional file 1: Text S1. Interview Guide [file 12913_2023_10219_MOESM1_ESM.docx]

# Text S1: Interview Guide

**Interview guide for focus group discussions and in-depth interviews**

(English version)

**I. General information**

1.1 Interviewer(s): _________________________ 1.2 Interviewee (unique code): _______

1.3 Date (example 12/07/2021): ___/___/_____ 1.4 Time of Interview: ___h/___min
1.5 Interviewee Profile (if in-depth interview): _____________________________________

1.6 District where the meeting is taking place: _____________________________________

**II. Questions**

1. Tell us about the actors (people) who are involved in the management of mental health problems in your setting, at all levels of care.

Relaunch:

a) Actors at the level of: the (sick) person (him/herself), the family, the community;

b) Caregivers in religious settings (religious); in neighborhoods (traditional healers); in health centers, general hospitals, private clinics (doctors, nurses); in psychiatric facilities (doctors…);

c) Of these actors that you mention, who do you think are currently active in mental health?

iv) How can we classify them?

2. Describe the tasks performed or roles played by the actors (that you mentioned above) including yourself in the management of mental health?

Relaunch:

a) The (sick) people themselves; b) Family members; c) Community members; d) Spiritual healers (or church-based healers); e) Traditional healers; f) Health center and general hospital providers (doctors, nurses, etc.); g) Psychiatric/mental health specialist providers (doctors, nurses, social workers, psychologists, etc.).

3. Tell us about any services (that you know of) that organize the care of the mentally ill in your health area/health district/town...

Relaunch:

a) Services that provide care in the informal sector and those that are official;

b) Of these services, are there any: Social services? Traditional health services? Primary care services? Specialized psychiatric services? Informal services that are difficult to classify?

4. How is the management of mental health and psychosocial support organized in the social and health services (structures) in your setting (health district/town...)?

Relaunch:

a) Which of these services: long-stay facilities, specialized mental health facilities, community-based mental health services, psychiatric inpatient services in general hospitals, mental health services through primary care services, informal community services; offer more care to people with mental health problems?

b) What about self-care?

c) Persons in charge of identification of a mental disorder; decision regarding a request for treatment; screening/diagnosis; decision to treat or refer; family and/or social support

d) Organization of traditional therapies and spiritual care or healing

5. What do you think about the collaboration modalities between care actors and what is called the "mix of services" of mental health care?

Relaunch:

a) Collaboration between "modern" and "traditional" care providers

b) Collaboration between formal services and informal structures

6. What are your expectations regarding mental health care and the organization (mix) of services?

7. What else do you have to say about our interview?

**Guide d’entretien pour les groupes de discussion et les entretiens approfondis**

(Version française)

**I. Informations générales**

1.1 Intervieweur(s) : _______________________ 1.2 Entretien (code unique) : _______

1.3 Date (exemple : 12/07/2021) : ___/___/_____ 1.4 Heure de l’entretien : ___h/___min
1.5 Profil de l’interviewé Profile (si entretien approfondi) : ___________________________

1.6 District où la réunion se tient : _______________________________________________

**II. Questions**

1. Parlez-nous des acteurs (personnes) qui sont impliqués dans la prise en charge de problèmes de santé mentale dans votre milieu, à tous les niveaux d’offre de soins.

Relances :

a) Acteurs au niveau de : la personne (malade lui/elle-même), la famille, de la communauté ;

b) Les soignants dans les milieux de cultes (religieux…) ; dans des quartiers (tradipraticiens…) ; dans des centres de santé, hôpitaux généraux, cliniques privées… (médecins, infirmiers…) ; dans des établissements de soins psychiatriques (médecins…) ;

c) De ces acteurs qui vous citez, qui pensez-vous être actuellement actifs dans la santé mentale ?

d) Comment peut-on les classifier ?

2. Décrivez les tâches exercées ou rôles joués par les acteurs (que vous avez cités précédemment) y compris vous-même dans la prise en charge de la santé mentale ?

Relances :

a) Les personnes (malades) elles-mêmes ; b) Les membres de famille ; c) Les membres de communauté ; d) Les religieux ; e) Les tradipraticiens ; f) Les prestataires de soins du centre de santé et des hôpitaux généraux (médecins, infirmiers…) ; g) Les spécialistes en psychiatrie / santé mentale (médecins, infirmiers, travailleurs sociaux, psychologues…).

3. Parlez-nous de tous services (que vous connaissez) qui organisent la prise en charge des malades mentaux dans votre aire de santé / district de santé / ville…

Relances :

a) Services qui soignent dans l’informel et ceux officiels ;

b) De ces services, existe-t-il de : Services sociaux ? Services de santé traditionnels ? Services de soins primaires ? Services spécialisés en psychiatrie ? Services informels difficiles à classifier ?

4. Comment s’organise la prise en charge de la santé mentale et soutien psychosocial dans les services (structures) sociaux et sanitaires dans votre milieu (district de santé / ville…) ?

Relances :

a) Lesquels d’entre ces services : établissements de long séjour, hôpitaux spécialisés en santé mentale, services de santé mentale à base communautaire, services psychiatriques dans les hôpitaux généraux, services communautaires informels ; offrent plus de soins aux personnes avec problèmes de santé mentale ?

b) Qu’en est-il des soins personnels (auto-soins) ?

c) Personnes en charge de : identification d’un problème de santé mentale ; décision concernant la demande de l’aide/traitement ; dépistage/diagnostic ; décision de traiter ou de référer ; soutien familial et/ou social ;

d) Organisation des soins de médecine traditionnelle et soins spirituels ou guérison spirituelle

5. Que pensez-vous des modalités de collaboration entre acteurs de prise en charge et de ce qu’on appelle « mix de services » de prise en charge de la santé mentale ?

Relances :

a) Collaboration entre les prestataires de soins « modernes » et « traditionnels »

b) Collaboration entre services officiels et structures informelles

6. Quelles sont vos attentes en matière de prise en charge en santé mentale et de l’organisation (mix) des services ?

7. Qu’avez-vous à ajouter à propos de notre entretien ?
