# Supplementary material for: Understanding the mix of services for mental health care in urban DR Congo: a qualitative descriptive study
Source: BMC Health Serv Res. 2023 Nov 4;23:1206. doi: 10.1186/s12913-023-10219-x (PMC10625694; doi:10.1186/s12913-023-10219-x)
Supplement: Supplementary file 2 — Additional file 2: Figure S1. Analytical framework of the mix of mental health services [file 12913_2023_10219_MOESM2_ESM.docx]

#
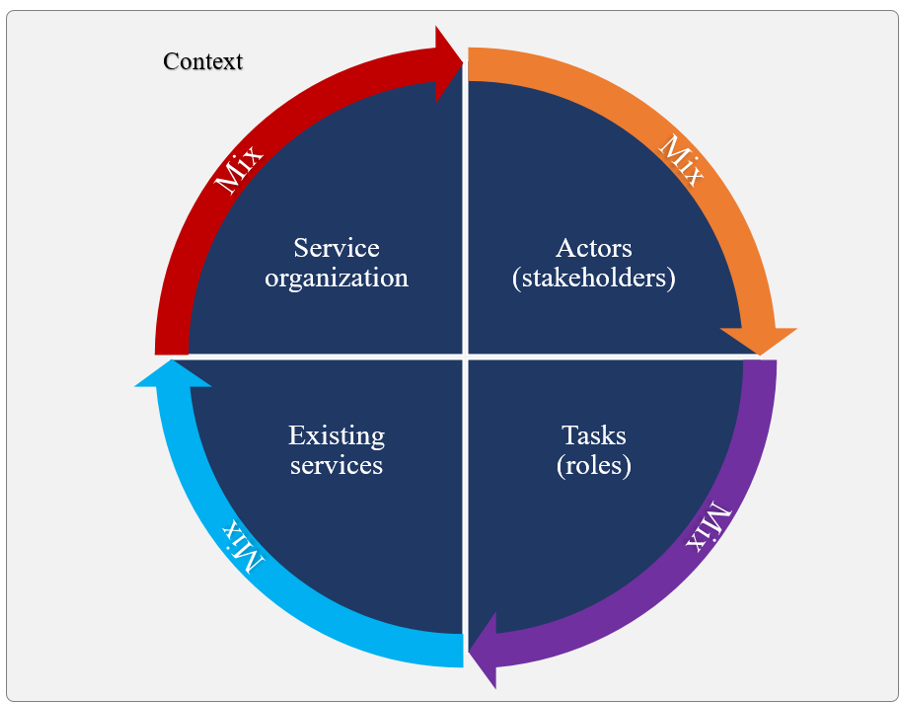
Figure S1: Analytical framework of the mix of mental health services

**Legend:**

- The ‘**Actors or stakeholders**’ theme includes the following sub-themes: patients (general population), caregivers, healers, and primary care providers.
- The ‘**Tasks or roles**’ theme includes the following sub-themes: identification of mental disorders, decision regarding a request for treatment, screening/diagnosis, decision to treat or refer, case management, psychosocial support, and self-care.
- The ‘**Existing services**’ theme includes the following sub-themes: informal services, social services, traditional therapy services, primary care services, and psychiatric facilities.
- The ‘**Service organization**’ theme includes the following sub-themes: collaboration between actors, and coordination of services.
- The ‘**Mix**’ theme refers to the following sub-themes: combination of services, combination of actors, combination of roles, combination of organizational aspects of services, location of services, and arrangement in the pyramid.
- **Context** refers to the working environment of the healthcare system.
